# A randomised controlled trial testing acceptance of practitioner‐referral versus self‐referral to stop smoking services within the Lung Screen Uptake Trial

**DOI:** 10.1111/add.16269

**Published:** 2023-06-18

**Authors:** Theodora Kotti, Evangelos Katsampouris, Mamta Ruparel, Andy McEwen, Jennifer L. Dickson, Stephen W. Duffy, Jo Waller, Samuel M. Janes, Samantha L. Quaife

**Affiliations:** ^1^ Research Department of Behavioural Science and Health University College London London United Kingdom; ^2^ Wolfson Institute of Population Health, Barts and The London School of Medicine and Dentistry Queen Mary University of London London United Kingdom; ^3^ Lungs for Living Research Centre, UCL Respiratory, Division of Medicine University College London London United Kingdom; ^4^ National Centre for Smoking Cessation and Training Dorchester United Kingdom; ^5^ School of Cancer and Pharmaceutical Sciences King’s College London London United Kingdom

**Keywords:** lung cancer, opt out, prevention, referral, screening, smoking cessation

## Abstract

**Background and aims:**

Optimising smoking cessation (SC) referral strategies within lung cancer screening (LCS) could significantly reduce lung cancer mortality. This study aimed to measure acceptance of referral to SC support by either practitioner‐referral or self‐referral among participants attending a hospital‐based lung health check appointment for LCS as part of the Lung Screen Uptake Trial.

**Design:**

Single‐blinded two‐arm randomised controlled trial.

**Setting:**

England.

**Participants:**

Six hundred forty‐two individuals ages 60 to 75 years, who self‐reported currently smoking or had a carbon monoxide reading over 10 ppm during the lung health check appointment.

**Intervention and comparator:**

Participants were randomised (1:1) to receive either a contact information card for self‐referral to a local stop smoking service (SSS) (self‐referral, *n* = 360) or a SSS referral made on their behalf by the nurse or trial practitioner (practitioner‐referral, *n* = 329).

**Measurements:**

The primary outcome was acceptance of the practitioner‐referral (defined as participants giving permission for their details to be shared with the local SSS) compared with acceptance of the self‐referral (defined as participants taking the physical SSS contact information card to refer themselves to the local SSS).

**Findings:**

Half (49.8%) accepted the practitioner‐made referral to a local SSS, whereas most (88.5%) accepted the self‐referral. The odds of accepting the practitioner‐referral were statistically significantly lower (adjusted odds ratio = 0.10; 95% confidence interval = 0.06–0.17) than the self‐ referral. In analyses stratified by group, greater quit confidence, quit attempts and Black ethnicity were associated with increased acceptance within the practitioner‐referral group. There were no statistically significant interactions between acceptance by referral group and any of the participants’ demographic or smoking characteristics.

**Conclusions:**

Among participants in hospital‐based lung cancer screening in England who self‐reported smoking or met a carbon monoxide cut‐off, both practitioner‐referral and self‐referral smoking cessation strategies were highly accepted. Although self‐referral was more frequently accepted, prior evidence suggests practitioner‐referrals increase quit attempts, suggesting practitioner‐referrals should be the first‐line strategy within lung cancer screening, with self‐referral offered as an alternative.

## INTRODUCTION

Tobacco smoking is the main risk factor for lung cancer in the United Kingdom (UK), the United States (US), China, Eastern European and Northern African countries; a disease that causes more deaths than any other type of cancer worldwide [1]. For individuals that smoke, smoking cessation (SC) remains the crucial preventive strategy for lung cancer, and for those with a long‐term smoking history, lung cancer screening (LCS) using low‐dose computed tomography (LDCT) can significantly reduce the risk of lung cancer mortality by detecting the disease early [2, 3]. Evidence suggests that the experience of LCS itself could increase motivation to quit [4], and it has been reported that the additive effect of smoking abstinence in the context of LCS increases the relative risk reduction from LCS in lung cancer mortality to 38% [5].

Those countries offering LCS programmes, therefore, mandate that SC support is offered to their high‐risk attendees. This includes people who currently smoke, 50 to 80 years of age, and with a significant long‐term smoking history. Identifying the most effective method for providing SC support is an ongoing area of research, but there are signals suggesting that the population might benefit from more proactive methods for accessing SC support than advice alone. Assessment of the 5As strategy (ask, advice, assess, assist and arrange) in the US National Lung Screening Trial (NLST) found that assisting and arranging SC support significantly increased the likelihood of quitting, compared with solely advising [6]. Smoking cessation advice and assistance by UK physicians to people who smoke were also found to be more effective strategy at increasing the success of quit attempts, rather than only advising people who smoke to stop or those being interested in quitting [7]. Similar active referral strategies were shown to be effective in midwifery research where ‘practitioner‐made’ (opt‐out) referral doubled self‐reported smoking cessation [8].

This trial compared rates and correlates of acceptance of a practitioner‐referral SC strategy relative to self‐referral SC strategy among people who currently smoke and are at high risk of lung cancer, within the UK LCS context.

## METHODS

### Design

This parallel, two‐arm, single‐blinded, between‐subjects, RCT assessed acceptance rates of a practitioner‐referral strategy, relative to a conventional self‐referral strategy, among people at high risk of lung cancer who currently smoke during a lung health check (LHC) appointment offering LDCT screening for lung cancer; and associations of acceptance for each referral strategy with demographic characteristics, smoking history, quit confidence and tobacco dependence.

### Participants

Individuals 60 to 75 years of age who had been recorded as smoking by their primary care practice within the previous 7 years were invited to a hospital‐based LHC appointment offering LCS as part of the Lung Screen Uptake Trial (LSUT) [9]. During this LHC appointment, participants (*n* = 689) who self‐reported currently smoking or had a carbon monoxide (CO) reading over 10 ppm (that are rare among those who have not smoked) were given ‘Very Brief Advice on Smoking’ [10]. A sample size of 289 participants in each arm was estimated to confer 90% power to detect a difference of between 10% and 20% with two‐sided testing and a 5% significance level (https://osf.io/ubech).

### Procedure

Using a web‐based programme, participants were randomised (1:1) to receive either a contact information card for self‐referral to a local stop smoking service (SSS) (self‐referral, *n* = 360) or a referral to a local SSS made on their behalf by the nurse or trial practitioner (practitioner‐referral, *n* = 329). For the practitioner‐referral, the nurse or trial practitioner identified the individual’s (geographically) closest SSS and referred them within 3 days using a brief, standardised electronic referral form sent to the respective SSS by email. Those declining the practitioner‐referral were offered the self‐referral contact information card as an alternative. Participants were unaware of this randomisation with each referral type presented as usual care.

### Measurements

Baseline demographic information was self‐reported during the LHC appointment (Table 1). The number of previous quit attempts and confidence in ability to quit smoking (from very low to extremely high) were also self‐reported during the LHC appointment [11]. Smoking status was collected through self‐reports and CO readings (≥10 ppm designated as a person who smokes). Tobacco dependence was measured by asking participants the time it took to start smoking after waking.

**TABLE 1:** Demographic and smoking characteristics of the analytical sample.

	Overall (*n* = 642)	Practitioner‐made referral group (*n* = 321)	Self‐referral group (*n* = 321)
Sex, % (*n*)			
Female	46.9 (301)	43.6 (140)	50.2 (161)
Male	53.1 (341)	56.4 (181)	49.8 (160)
Age, *M* years (*SD*)	66.0 (4.2)	66.1 (4.2)	65.8 (4.2)
Ethnicity, % (*n*)			
White	81.9 (526)	82.2 (264)	81.6 (262)
Asian	2.0 (13)	1.2 (4)	2.8 (9)
Black	11.1 (71)	11.2 (36)	10.9 (35)
Mixed	1.1 (7)	1.2 (4)	0.9 (3)
Other	3.4 (22)	3.4 (11)	3.4 (11)
Not stated	0.5 (3)	0.6 (2)	0.3 (1)
IMD rank, % (*n*)^a^			
Quintile 1 (1–6496) most deprived	55.1 (354)	54.8 (176)	55.5 (178)
Quintile 2 (6497–12 993)	34.0 (218)	34.9 (112)	33.0 (106)
Quintile 3 (12 994–19 489)	1.6 (10)	1.6 (5)	1.6 (5)
Quintile 4 (19 490–25 986)	0.3 (2)	0.0 (0)	0.6 (2)
Quintile 5 (25 987–32 482) least deprived	0.0 (0)	0.0 (0)	0.0 (0)
Missing	9.0 (58)	8.7 (28)	9.3 (30)
Previous quit attempts, % (*n*)			
None	19.3 (124)	19.3 (62)	19.3 (62)
1–4	60.9 (391)	63.9 (205)	57.9 (186)
>5	19.6 (126)	16.5 (53)	22.7 (73)
Missing	0.2 (1)	0.3 (1)	0.0 (0)
Pack years, *M* (SD)	40.7 (25.3)	40.4 (25.4)	40.9 (25.2)
Quit confidence, % (*n*)			
Very low	20.6 (132)	19.3 (62)	21.8 (70)
Low	15.0 (96)	14.0 (45)	15.9 (51)
Not very high	23.2 (149)	24.0 (77)	22.4 (72)
Quite high	20.1 (129)	19.3 (62)	20.9 (67)
Very high	11.7 (75)	11.2 (36)	12.1 (39)
Extremely high	7.6 (49)	9.7 (31)	5.6 (18)
Missing	1.9 (12)	2.5 (8)	1.2 (4)
Carbon monoxide reading in parts per million, mean (range)	14.0 (1–53)	14.1 (1–51)	13.8 (1–53)
Time to first cigarette, % (*n*)			
Within 5 min	16.5 (106)	16.5 (53)	16.5 (53)
6–30 min	32.9 (211)	29.0 (93)	36.8 (118)
31–60 min	17.9 (115)	17.4 (56)	18.4 (59)
>60 min	31.6 (203)	35.8 (115)	27.4 (88)
Proportion accepting referral, % (*n*)			
Acceptance	69.2 (444)	49.8 (160)	88.5 (284)
Refusal	30.8 (198)	50.2 (161)	11.5 (37)

*Note*: % totals may not sum because of rounding.

^a^ IMD rank: English national Index of Multiple Deprivation Quintile (2015).

### Statistical analysis

The primary outcome was acceptance of each referral type (i.e. practitioner‐referred participants giving permission for their details to be shared with the local SSS or participants taking the physical SSS contact information card to refer themselves). This deviates from the prospectively published statistical analysis plan (https://osf.io/ubech/) because it was not possible to collect data on attendance and quit attempts from SSS because of changes in the commissioning and providers of these services during the period of data collection. The absolute proportions and correlates of acceptance for each SC referral type were assessed using descriptive statistics and logistic regression analyses adjusted for demographic (i.e. sex, age, ethnicity and Index of Multiple Deprivation [IMD] rank), smoking and quit history, CO reading and tobacco dependence. Interaction terms were then examined between referral group and each of the demographic and smoking variables.

## RESULTS

The sample, had a mean age of 66.0 years (SD = 4.2), were predominantly male (53.1%), of a White ethnic background (81.9%), living within the most deprived quintile nationally (55.1%) and had an average smoking history of 40.7 pack years (SD = 25.3). Most (80.5%) had tried to quit smoking previously, with 58.8% reporting very low, low or not very high quit confidence (Table 1). The mean exhaled CO reading was 14.0 ppm, with half (49.4%) the sample reporting smoking within 30 minutes of waking. The analytical sample (*n* = 642) excluded 47 cases because of randomisation mistreatment with implausible acceptance data compared with their allocated group (recorded as accepting both referral types [*n* = 7], accepting the self‐referral twice [*n* = 12], both accepting and refusing the self‐referral [*n* = 27] or missing [*n* = 1]).

In the practitioner‐referral group, 49.8% (160/321) accepted the practitioner‐referral to an SSS, and in the self‐referral group, 88.5% (284/321) accepted a card with details for self‐referral to an SSS. The odds of accepting the practitioner‐referral were statistically significantly lower (adjusted odds ratio [aOR] = 0.10; 0.06–0.17) than the self‐referral (Table 2).

**TABLE 2:** The association between demographic and smoking characteristics and acceptance (vs refusal) of the practitioner‐made or self‐referral smoking cessation strategy.

	Overall (*n* = 642), aOR (95% CI)	Practitioner‐made referral group (*n* = 321), aOR (95% CI)	Self‐referral group (*n* = 321), aOR (95% CI)
Sex	*P* = 0.027	*P* = 0.168	*P* = 0.112
Female	Ref.	Ref.	Ref.
Male	0.61 (0.40–0.95)*	0.69 (0.41–1.17)	0.50 (0.21–1.18)
Age	*P* = 0.260 0.97 (0.92–1.02)	*P* = 0.349 0.97 (0.91–1.03)	*P* = 0.742 0.98 (0.89–1.09)
Ethnicity	*P* = 0.057	*P* = 0.071	*P* = 0.665
White	Ref.	Ref.	Ref.
Asian/Mixed/other	1.25 (0. 52–2.98)	1.31 (0.48–3.55)	0.82 (0.15–4.53)
Black	2.70 (1.19–6.10)*	3.03 (1.18–7.79)*	2.05 (0.39–10.69)
IMD rank^a^	*P* = 0.422	*P* = 0.153	*P* = 0.391
Quintile 1 (most deprived)	Ref.	Ref.	Ref.
Quintile 2, 3, 4	1.20 (0.77–1.87)	1.20 (0.70–2.05)	1.49 (0.60–3.66)
Previous quit attempts	*P* = 0.002	*P* = 0.023	*P* = 0.095
None	Ref.	Ref.	Ref.
1–4	1.84 (1.07–3.14)*	1.78 (0.91–3.48)	1.89 (0.73–4.90)
>5	3.58 (1.77–7.26)*	3.39 (1.42–8.09)*	4.27 (1.14–16.07)*
Pack years	*P* = 0.989 1.00 (0.99–1.01)	*P* = 0.430 1.00 (0.99–1.02)	*P* = 0.257 0.99 (0.98–1.01)
Quit confidence	*P* = 0.002	*P* = 0.057	*P* = 0.110
Very low	Ref.	Ref.	Ref.
Low	1.40 (0.70–2.77)	1.18 (0.49–2.87)	1.74 (0.54–5.59)
Not very high	3.28 (1.72–6.26)*	2.64 (1.20–5.78)*	8.31 (1.74–39.76)*
Quite high	2.57 (1.30–5.08)*	2.58 (1.12–5.93)*	2.41 (0.72–8.07)
Very high	2.63 (1.27–6.31)*	2.55 (0.95–6.88)	3.31 (0.76–14.46)
Extremely high	1.15 (0.49–2.74)	1.09 (0.38–3.13)	1.23 (0.26–5.82)
Carbon monoxide reading	*P* = 0.012 1.04 (1.01–1.07)*	*P* = 0.019 1.04 (1.01–1.08)*	*P* = 0.250 1.04 (0.98–1.10)
Time to first cigarette	*P* = 0.378	*P* = 0.367	*P* = 0.725
Within 5 min	Ref.	Ref.	Ref.
6–30 min	1.63 (0.86–3.09)	1.57 (0.71–3.47)	1.75 (0.54–5.71)
31–60 min	1.76 (0.84–3.71)	2.25 (0.91–5.60)	1.03 (0.27–3.87)
>60 min	1.34 (0.65–2.73)	1.51 (0.62–3.65)	1.04 (0.29–3.81)
Referral group	*P* < 0.001		
Self‐referral	Ref.	–	–
Practitioner‐made	0.10 (0.06–0.17)*	–	–

Abbreviation: aOR, adjusted odds ratio.

^a^ IMD rank: National Index of Multiple Deprivation Quintile (2015).

* *P* < 0.05 for sub‐category associations.

In the practitioner‐referral group, compared to those who had ‘never attempted to quit’ smoking, those who had attempted to quit previously (e.g. >5 attempts) were more likely to accept the practitioner‐referral to an SSS (aOR = 3.39; 1.42–8.09). Compared to those self‐reporting ‘very low’ confidence to quit smoking, those self‐reporting ‘not very high’ and ‘quite high’ confidence to quit were more likely to accept the practitioner‐referral (aOR = 2.64; 1.20–5.78 and aOR = 2.58; 1.12–5.93, respectively). A higher exhaled CO reading was also associated with acceptance (OR = 1.04; 1.01–1.08), as was ethnicity, with those reporting their ethnicity as Black being more likely to accept the practitioner‐referral than those of a White ethnic background (aOR = 3.03; 1.18–7.79).

In the self‐referral group, compared to those who self‐reported ‘very low’ confidence to quit smoking, the odds of accepting a self‐referral to an SSS were higher for those who self‐reported ‘not very high’ confidence to quit (aOR = 8.31; 1.74–39.76).

A higher CO reading increased the odds of accepting a SSS referral overall (aOR = 1.04; 1.01–1.07), but there was no interaction with referral group. Sex, age, deprivation, pack‐year history and time to first cigarette were not associated with acceptance of either referral strategy (Table 2) nor were there any interactions between these characteristics and referral group in predicting referral acceptance.

## DISCUSSION

This RCT examined the rates of acceptance for a practitioner‐referral SC strategy when compared with a conventional self‐referral approach among people at high risk of lung cancer who currently smoke during a LHC appointment offering LDCT screening for lung cancer. Although acceptance of the self‐referral was greater than the practitioner‐referral (88.5% vs. 49.8%), it was high for both strategies indicating high receptiveness to SC support among lung screening attendees. Evidence has shown that practitioner‐made referral approaches increase quit attempts and smoking abstinence in pregnant women and intensive cessation intervention in lung screening increase short‐term quit rates [8, 12], although low quit rates were found in a telephone‐based smoking cessation counselling intervention embedded into LCS [13]. Our findings, interpreted with caution, suggest practitioner‐referrals as an acceptable first‐line strategy for arranging SC support for people who currently smoke attending LCS, in particular for individuals of a Black ethnic background, and when self‐referral is offered as an alternative to those declining the practitioner‐referral.

Greater confidence and experience of trying to quit smoking were associated with higher odds of acceptance for both referral types. Those individuals who feel less motivated or less able to quit may need more intensive and individualised interventions to support engagement with SC support. This may include a co‐located SC advisor and personalised intervention, as is being trialled by the Yorkshire Enhanced Smoking Cessation Study [14]. Assessing levels of confidence to quit and quit history before the intervention could also help personalise the type or intensity of support offered. In the meantime, a practitioner‐referral approach is seen as feasible, scalable and a ‘more ethical’ choice to increase uptake of effective SC strategies [15].

The finding that those from Black ethnic backgrounds, specific to the UK setting, were more likely to accept the practitioner‐referral than those from White ethnic backgrounds is interesting [9]. Because of lack of evidence in this area, we are only able to speculate about the possible reasons for this. Although people of a Black ethnic background might be less likely to succeed in quitting smoking in the long term than those of a White ethnic background who smoke, evidence shows they make relatively more attempts to quit [16, 17]. Therefore, in the present study their increased likelihood to accept the practitioner‐referral specifically could plausibly reflect higher motivation for or receptivity to, proactive methods of support. Other studies suggest those from ethnic minority backgrounds may be less likely to receive SC support to quit from health professionals [18], which universal approaches like practitioner‐referral strategies, could help to overcome. Further research is needed to understand the reliability of this finding, and if so, understand the reasons for differences in acceptance of this proactive referral approach.

We were unable to examine subsequent SSS attendance so it remains unclear if individuals pursued either type of referral and whether there were differences in uptake or quit rates between arms. Although acceptance of the self‐referral was higher, fewer may have subsequently engaged with a SSS, and arguably, acceptance of the self‐referral requires a lesser immediate commitment from the individual. However, evidence suggests that if individuals do engage with SSS support, their chances of quitting could increase threefold [10]. Indeed, arranging SC support in the US NLST significantly increased the odds of quitting [6]. Additionally, opt‐out referral strategies, as recommended by UK government guidelines, have been seen as a potentially acceptable addition to midwifery practice, which may increase motivation to quit smoking, with self‐referral strategies predicting increased acceptability and engagement with SC support in later pregnancy [19, 20]. Further research is needed to understand uptake of SSS support following practitioner‐referrals in a LCS setting, as well as subsequent quit attempts.

Despite the application of a RCT to compare acceptance rates for a practitioner‐referral with those for a self‐referral among those at high risk of lung cancer who currently smoke, the present findings should be regarded as suggestive rather than conclusive, and we acknowledge a number of limitations that future research needs to address. A major limitation was the inability to measure individuals’ subsequent attendance at and engagement with SSS, as previously discussed [6, 8]. Another limitation is the generalisability of findings. Although the LSUT recruited individuals from several socio‐economic backgrounds to ensure sample diversity and external validity, the current sample consisted mostly of people from a White ethnic background living within the most deprived areas and a narrower age‐range compared with national lung screening programmes. Last, although the absolute number of excluded cases with implausible data was small, this further limited study analysis.

In conclusion, this RCT found relatively high acceptance rates for both referrals, suggesting that LCS provides the opportunity to support people with long‐term smoking at high risk who are receptive to support. Given accumulating evidence for the effectiveness of practitioner‐made referral approaches in increasing the odds of quitting smoking, we recommend a practitioner‐referral strategy as a minimum standard of care for LCS programmes, implemented as a first‐line strategy with self‐referral offered as a second‐line alternative to those declining the practitioner‐referral. Future research should examine the effectiveness of practitioner‐referral SC strategies in increasing quit rates specifically within the LCS setting as well as developing more personalised approaches for engaging those less able to quit smoking.

## AUTHOR CONTRIBUTIONS

**Theodora Kotti:** Data curation (equal); formal analysis (equal); visualization (equal); writing—original draft (equal); writing—review and editing (equal). **Evangelos Katsampouris:** Visualization (equal); writing—original draft (equal); writing—review and editing (lead). **Mamta Ruparel:** Conceptualization (equal); data curation (equal); investigation (equal); methodology (equal); writing—review and editing (equal). **Andy McEwen:** Conceptualization (equal); funding acquisition (equal); investigation (equal); methodology (equal); writing—review and editing (equal). **Jennifer L. Dickson:** Data curation (equal); investigation (equal); writing—review and editing (equal). **Stephen W. Duffy:** Conceptualization (equal); funding acquisition (equal); methodology (equal); writing—review and editing (equal). **Jo Waller:** Conceptualization (equal); methodology (equal); writing—review and editing (equal). **Samuel M. Janes:** Funding acquisition (equal); investigation (equal); methodology (equal); writing—review and editing (equal). **Samantha L. Quaife:** Conceptualization (equal); data curation (equal); funding acquisition (equal); investigation (equal); methodology (equal); supervision (equal); writing—original draft (equal); writing—review and editing (lead).

## DECLARATION OF INTERESTS

T.K., E.K., A.M., J.W., S.W.D. and S.L.Q. have no conflicts of interest to declare. S.M.J. is the Chief Investigator for an academic study (SUMMIT), which is sponsored and conducted by UCL and funded by GRAIL, through a research grant. J.L.D. is, and M.R. has previously been, supported as investigators by this funding. S.M.J. has been paid by Astra Zeneca, BARD1 Bioscience, Jansen and Achilles Therapeutics for being an Advisory Board Expert and travel to one US conference. S.M.J. receives grant funding from Owlstone for a separate research study. M.R. received travel funding for a conference from Takeda and an honorarium for planning and speaking at educational meetings from Astra Zeneca. All authors perceive that these disclosures pose no academic conflict for this study. All authors declare no other relationships or activities that could appear to have influenced the submitted work.

## Data Availability

Data available on request due to privacy/ethical restrictions.
